# Antimicrobial Hydrophilic Membrane Formed by Incorporation of Polymeric Surfactant and Patchouli Oil

**DOI:** 10.3390/polym13223872

**Published:** 2021-11-09

**Authors:** Nasrul Arahman, Cut Meurah Rosnelly, Diki Sukma Windana, Afrillia Fahrina, Silmina Silmina, Teuku Maimun, Sri Mulyati, Umi Fathanah, Sri Aprilia, Muhammad Roil Bilad, Poernomo Gunawan, Wafiq Alni Dzulhijjah, Nur Halimah

**Affiliations:** 1 Department of Chemical Engineering, Universitas Syiah Kuala, Banda Aceh 23111, Indonesia; cut.meurah@che.unsyiah.ac.id (C.M.R.); dikiwindana@gmail.com (D.S.W.); afrilliafahrina26@gmail.com (A.F.); Minaelsilmina@gmail.com (S.S.); maimunteuku@gmail.com (T.M.); sri.mulyati@unsyiah.ac.id (S.M.); umifathanah@unsyiah.ac.id (U.F.); sriaprilia@unsyiah.ac.id (S.A.); wafiqalnidz@gmail.com (W.A.D.); Nhalimahh.23@gmail.com (N.H.); 2 Magister Program of Environmental Management, Universitas Syiah Kuala, Banda Aceh 23111, Indonesia; 3 Research Center for Environmental and Natural Resources, Universitas Syiah Kuala, Jl. Hamzah Fansuri, No. 4, Darussalam, Banda Aceh 23111, Indonesia; 4 Atsiri Research Center, PUI, Universitas Syiah Kuala, Banda Aceh 23111, Indonesia; 5 Faculty of Integrated Technologies, Universiti Brunei Darussalam, Bandar Seri Begawan BE1410, Brunei; roil.bilad@ubd.edu.bn; 6 School of Chemical & Biomedical Engineering, Nanyang Technological, University Singapore, Singapore 637459, Singapore; pgunawan@ntu.edu.sg

**Keywords:** polyethersulfone, pluronic, patchouli, pesticide, blending, ultrafiltrasi, cross-flow, membrane, separation

## Abstract

Membrane properties are highly affected by the composition of the polymer solutions that make up the membrane material and their influence in the filtration performance on the separation or purification process. This paper studies the effects of the addition of pluronic (Plu) and patchouli oil (PO) in a polyethersulfone (PES) solution on the membrane morphology, membrane hydrophilicity, and filtration performance in the pesticide removal compound in the water sample. Three types of membranes with the composition of PES, PES + Plu, and PES + Plu + patchouli oil were prepared through a polymer phase inversion technique in an aqueous solvent. The resulting membranes were then analyzed and tested for their mechanical properties, hydrophilicity, antimicrobial properties, and filtration performance (cross-flow ultrafiltration). The results show that all of the prepared membranes could reject 75% of the pesticide. The modification of the PES membrane with Plu was shown to increase the overall pore size by altering the pore morphology of the pristine PES, which eventually increased the permeation flux of the ultrafiltration process. Furthermore, patchouli oil added antimicrobial properties, potentially minimizing the biofilm formation on the membrane surface.

## 1. Introduction

The selection of constituent materials for membrane development focuses on producing membranes with strong mechanical properties, good anti-fouling ability, and high chemical resistance. To achieve these ideal properties, a polymer is often modified with other materials to produce a membrane with optimal filtration performance [1,2,3,4]. One of the most common techniques is blending the polymer with hydrophilic additives in the dope solution [5,6,7]. Besides acting as a structure modifying agent, the additive material can minimize the propensity of biofouling during the filtration process by increasing the membrane surface hydrophylicity. Alternatively, post-treatment through membrane surface grafting with certain monomers can also be employed [8,9,10,11], or surface coating by a specific polymers [12].

Biofouling is typically caused by a biofilm formation of microorganisms on the membrane surface [13]. Biofouling can be a serious problem because it can block the pores and therefore reduce the membrane filtration performance. Consequently, it shortens the membrane lifespan and increases the operating costs due to frequent chemical cleaning [14,15,16]. Therefore, additives with a good antimicrobial property that can prevent bacterial activity on the membrane surface are needed.

One way to prevent biofouling is by adding antimicrobial substances into the membrane matrix, such as titanium dioxide (TiO_2_) nanoparticles, nanosilver (Ag), nano-sized hydrous manganese dioxide (HMO), and graphene oxide (GO) nanoparticles [17,18]. A research group led by Qiblawey investigated the effect of blending a polysulfone (PSf) poymer with a treated GO on the antibacterial activity of the PSf blend membrane [19]. The experimental results show that nanosilver can kill up to 83.6% of bacteria on the membrane surface and thus prevent a rapid decrease in permeate flux. Kumar et al. added an antimicrobial additive derived from biomass, namely curcumin [20]. The authors confirmed that reducing microbe life on the casted membrane upgraded the hydrophilicity membrane properties. Water permeability was shown to have been enhanced significantly, reaching 88.52%. Moreover, the addition of nano curcumin also increased the membrane mechanical strength by 58%. Biomass-derived materials offer some advantages over their synthetic counterparts as the former are renewable and more economical, hence more sustainable.

**Figure 1:**
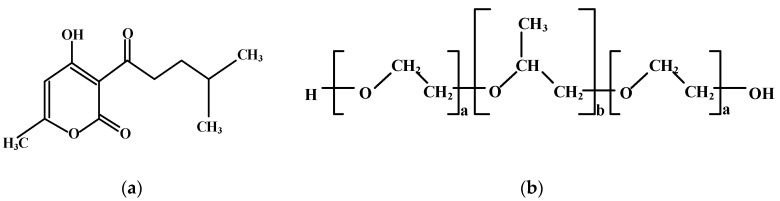
Chemical structure of (**a**) PO (Pogostone), and (**b**) Pluronic F127.

In this study, the improvement of the PES membranes’ anti-bacterial properties was carried out by adding patchouli oil (PO) from patchouli plant extract (*Pogostemon cablin Benth Patchouli*). The use of PO as a membrane additive is strongly supported by the availability of patchouli plants in Indonesia, especially in Aceh Province, the largest producer of patchouli plants globally. PO as a general chemical structure, as shown in Figure 1a, contains terpene compounds such as aromatic and phenolic compounds, which are known to be anti-bacterial, antifungal, and antioxidant [21]. Therefore, it is expected to minimize biofouling and thereby prolong the lifespan of the PES membrane. Despite numerous studies that have been reported enhancing the antibacterial properties of ultrafiltration membranes, as far as the literature is concerned, this is the first attempt to use PO for improving the properties and filtration performance of PES membranes. To further increase the surface hydrophilicity, porosity, and membrane pore size, a polymeric surfactant pluronic F127 (Plu) was also introduced in the polymer solution (Figure 1b). The combination of Plu and PO is envisaged to give a synergistic effect to improve the hydrophilicity property, water permeation, and membrane lifetime during the ultrafiltration processes.

## 2. Materials and Methods

### 2.1. Materials

To prepare the polymeric dope solution, polyethersulfone (PES Ultrason E6020, 99%, BASF, Ludwigshafen, Germany), Pluronic F127 (99%, WAKO, Osaka, Japan), and patchouli oil (Atsiri research center, USK, Banda Aceh, Indonesia) were dissolved in dimethyl formamide (DMF 99.8%, -Sigma Aldrich, Burlington, MA, United States). Deionized water (ultrapure milli-Q) was used for membrane solidification. Fipronil pesticide (50 g/L, BASF, Ludwigshafen, Germany) was used as a model compound to test the ultrafiltration process and evaluate the membrane rejection property. Escherichia coli (ATCC 25922) and Mueller–Hinton agar (OXOID, Ottawa, Canada) were employed to evaluate anti-bacterial activity of membrane.

### 2.2. Membrane Fabrication

Three kinds of polymer solutions were prepared to fabricate three types of membranes using DMF as the solvent. The proportions of all prepared membranes have been summarized (shown in Table 1). A magnetic stirrer plate was used to stir the solution for 24 h at room temperature to achieve a homogeneous and bubble-free blend polymer solution. After ensuring that the polymer mixture solution’s air bubbles had completely disappeared, the polymer was printed on a glass plate using an automatic membrane applicator, a Yoshimitsu device from Japan, to achieve a 200 µm thickness. Next, a deionized water coagulation vessel was used to immerse the glass plate and cast film as a non-solvent phase inversion. The casted membrane was then rinsed in water to eliminate residual solvent before it was characterized.

### 2.3. Membrane Properties and Morphology

The membrane mechanical properties (in terms of the tensile test) were evaluated using the Autograph AGS J device, Japan, in line with ASTM D 638-14. A piece of membrane fragment (5 cm length) was installed on the tensile tool panel. The tensile strength value of each membrane was obtained from the average of 5 analyses. A goniometer (Drop Master 300, Kyowa Interface Science Co., Japan) was used to determine the water drop contact angle to analyze hydrophilic membrane properties. Five microliters of water was dropped onto the membrane surface through a microneedle. The angle of the water droplet atop the surface was automatically recorded. The measurements were done 10 times for each sample, and the results are presented as average. A scanning Electron Microscopy (SEM JSF-7500F, JEOL, Japan) was used to assess the membrane surface and cross-section morphologies. For the cross-sectional structure, a piece of the membrane was immersed and crushed into the liquid nitrogen. All membranes were coated with osmium powder before scanning, and then underwent the scanning process. The structure of the membrane in terms of surface roughness was analyzed using atomic force microscopy (AFM) with the method described previously [22,23]. The functional groups of each membrane near the surface were identified using a Fourier Transform Infrared Spectroscopy (FTIR spectrophotometer, Thermo Scientific, Japan). The drying of each membrane sample of 1 cm^2^ at 40 °C was carried out for 1 h. This process aims to remove the moisture content before analysis. Then the membrane was installed on the sample panel, and at the same time, the panel recorded the absorbance spectrum in the maximum wavenumber range.

### 2.4. Anti-Bacterial Test

In this study, the performance of the membrane in preventing biofouling was analyzed by the investigated anti-bacterial activity. The anti-microbial test was carried against Gram-negative bacteria, Escherichia coli (ATCC 25922). A 0.5 Mac Farland *of E. coli* solution was swabbed onto Mueller–Hinton agar in a petri dish. Then, the sterile membrane sample was put on the surface of agar containing of *E. coli.* The number of colonies that grew on the membrane surface were counted under a magnifying glass after 24 h of incubation.

### 2.5. Permeation Test

**Figure 2:**
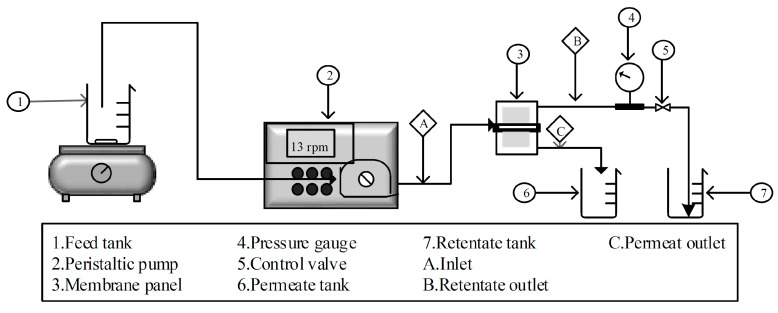
Cross-flow filtration system used for the filtration performance testing.

In determining the membrane flux, a cross-flow filtration module can be used (illustrated in Figure 2). Distilled water was fed to the cross-flow module at 0.1 L min^−1^ flow rate with an applied pressure of 1.0 bar through the peristaltic pump. Filtration time recording started when the water exited through the membrane wall, and the measurements of the permeate flow rate were taken every 10 min starting from when the filtration began until it reached a constant flux. The membrane flux was calculated using Equation (1). The membrane flux measurement was repeated 3 times, and the average results from 3 trials were taken as membrane filtration performance data.

(1) $$J_{m}=\frac{V_{p}}{A\times \Delta t}.$$

where J_m_, V_p_, Δt, and A are the water flux (L m^−2^·h^−1^), volume of the filtrate (L), and the effective area of membrane (m^2^), respectively.

### 2.6. Pesticide Removal Test

The fipronil standard solution (BASF) was used as a model compound for the pesticide removal test. An amount of 1 mL of fipronil at a concentration of 50 g/L was dissolved in 10 mL of 99% ethanol. The resulting solution was further dissolved in 1000 mL of distilled water to make a 50 ppm fipronil solution. The pesticide removal test was carried out with the same cross-flow ultrafiltration module used for the permeation test (Figure 1). The final concentration of the pesticide solution was analyzed using a spectrophotometer (Shimadzu Spectrophotometer UV-1800, Kyoto, Japan). The rejection of the pesticide (Equation (2)) was evaluated by measuring the difference between the initial and final fipronil concentrations (feed and permeate solutions), respectively.

(2) $$R_{m}=\left(1-\frac{C_{p}}{C_{f}}\right)\times 100\%$$

where R_m_ is the rejection of the pesticide (%), and C_p_ and C_f_ are the fipronil content in the residue and filtrate (mg L^−1^), respectively.

## 3. Results and Discussion

### 3.1. Membrane Structure

**Figure 3:**
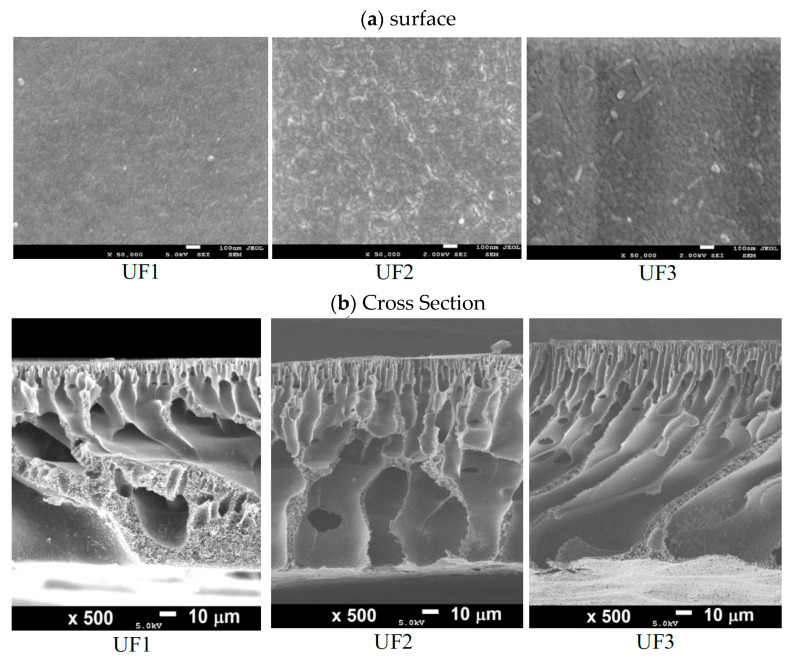
SEM images of the prepared membranes: (**a**) membrane surface and (**b**) membrane cross-section.

The mechanism of fabricated membranes was affected by the non-solvent induced phase separation (NIPS) method based on mass transfer. Mass transfer with non-solvent reveals a substantial impact on obtaining porous asymmetric membranes [24]. The NIPS method results in a dense skin layer and finger-like cross-section on the prepared membrane [25]. The structure of membranes followed the NIPS method when the coagulation water bath was at room temperature, as illustrated in Figure 3.

**Figure 4:**
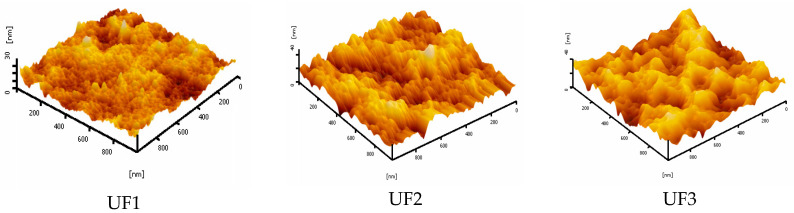
AFM images of the membranes surface.

Figure 3 shows the surface and structure of the cross-section of all prepared membranes. It can be seen in Figure 3a that the surface of the UF1 membrane (pristine PES) had a dense structure with small nodules without pores. Blending PES polymer with 3 wt % Plu resulted in a membrane with a porous surface, with a pore size of less than 50 nm (UF2). Plu is a surfactant polymer that may act as a membrane pore-forming agent through an irregular bonding scheme between the primary polymer and additive particles. Due to the bond irregularity, the space between the particles opens, forming membrane pores. Several studies reported that Plu as a membrane-modifying agent has successfully changed the pore structure in membranes made of various polymers [7,26,27]. In particular, the surface of the UF3 membrane depicts an aggregation of Plu and PO particles. The dispersion of these aggregates/clusters of PO and Plu particles is related to the physical group of polymer chains [28]. The detail surface morphology of the membrane might also be confirmed by the roughness data obtained from AFM analysis. Figure 4 shows the 3D AFM image of the membrane surface, in which the surface roughness has clearly increased (Ra). It is confirmed that the Ra of the UF1, UF2, and UF3 membranes are 2.04, 4.75, and 4.92, respectively.

The observation on the membrane cross-sectional structure further reveals the effect of Plu and PO additions on membrane morphology (Figure 3b). In general, all of the prepared membranes exhibited a dense selective layer at the top, which is supported by the microporous layer underneath. It is evident that the addition of Plu (UF2) and PO (UF3) created large, finger-like macrovoids, whereas the pristine PES membrane (UF1) is dominated by sponge-like structures.

The results indicate that modification with additive materials affects the formation of new pores and minimizes the dense structure of the PES membrane. The addition of PO and Plu led to instant segregation during the phase separation in the coagulation bath. This phenomenon occurs due to the unstable dope solution from a thermodynamic perspective. The thermodynamics of the solvent-non-solvent system, polymer-patch/Plu, and the segregation process kinetics significantly affect the themorphology of the formed membranes. Furthermore, there is aggregation between PES polymers and PO and Plu Particles, which removes the solvent from the system during the precipitation process in the coagulation bath, and the result is an enlarged pore size that looks like a macro-finger void. The large macro voids’ formation resulted from instant segregation in which the solvent entered the membrane film through the crack formed by the skin-layer crack [28,29].

### 3.2. Chemical Compounds Indication

**Figure 5:**
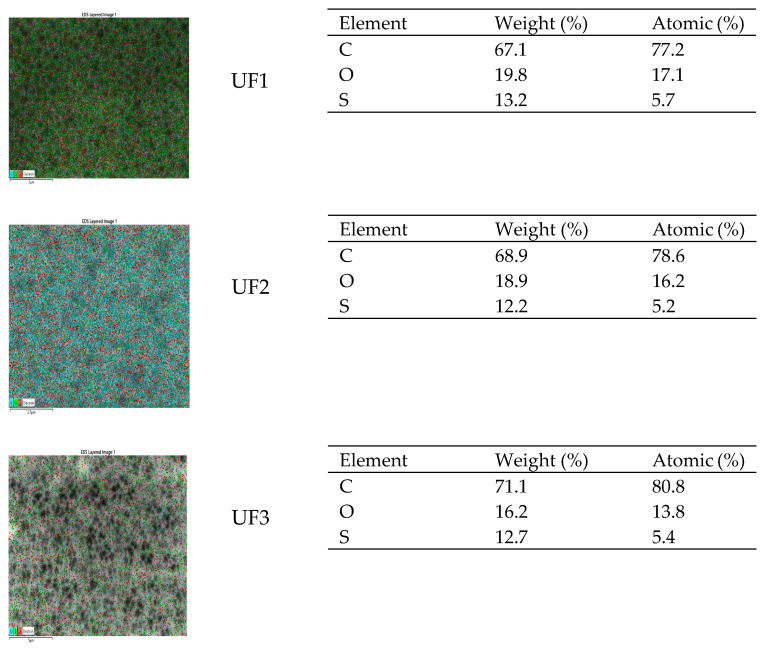
**EDXS** images of the membranes.

An energy-dispersive X-ray spectroscopy (EDXS) test was conducted to analyze the elemental composition of the membrane surface. Figure 5 shows the EDXS spectrum of the PES membrane (UF1), the PES membrane modified with Plu 3% (UF2), and the PES membrane modified with Plu 3% and PO 3% (UF3). The result reveals that carbon (C), oxygen (O), and sulfur (S) are present on all membrane surfaces. Similarly, Elcik et al. reported that C, O, and S compositions were obtained in the PES membrane [30]. By adding the pluronic and patchouli oil, the ratio of carbon atoms increases; however, oxygen and sulfur atoms decrease (Figure 5).

**Figure 6:**
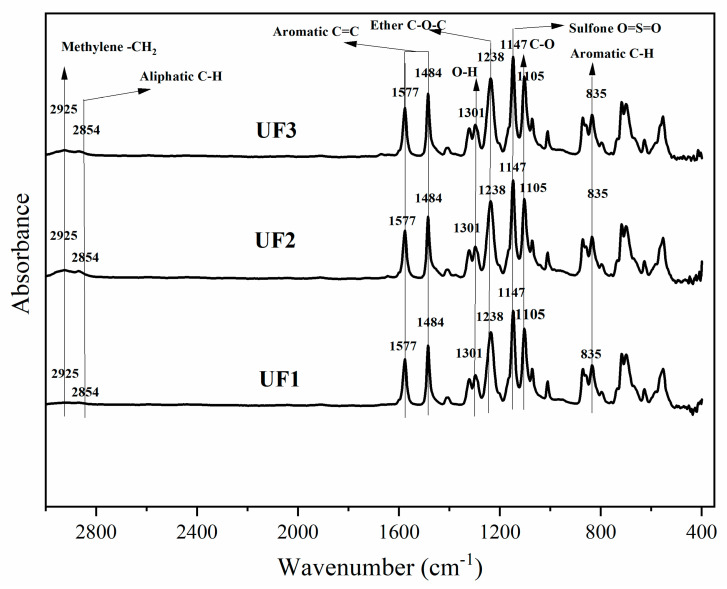
IR spectra of the prepared membranes.

The results of the FTIR analysis for all samples are shown in Figure 6. All samples contain aromatic ring groups (C=C) that appear at 1484 and 1577 cm^−1^. The sulfone groups (O=S=O) were detected at 1147 cm^−1^, while aromatic ether groups (C-O-C) appeared at a 1238 cm^−1^. Furthermore, aromatic C-H groups were detected at 835 cm^−1^. All of these spectra are related to the presence of atomic vibrations in the material, which is a characteristic of PES.

The Plu functional clusters (C-O, O-H, and C-H) were detected at 1105, 1301, and 2854 cm^−1^, respectively. However, pure PO-characterizing groups, as reported by Fahmi et al. [31], such as *cis* (C-CH), *methyl* (-CH_3_), *methylene* (-CH_2_), *carbonyl* (C=O), *cis* (C=C), *alcohol* (C-O), *ether* (C-O), *trans* (-HC=CH), and *cis* (-HC=CH) are hardly noticeable in this membrane. This phenomenon is presumed to be caused by leaching that most of the PO particles undergo during the phase inversion process. It is worth noting that the peaks at 1105 and 1301 cm^−1^ cannot be exclusively attributed to Plu. They also corresponded to the C-H bending and C-O stretching vibration that were available in the PES structure, as such both peaks also presented in the UF1 membrane.

### 3.3. Hydrophilic Properties

The hydrophilic surface is an essential part in improving the membrane permeability and properties of anti-fouling [32]. Meanwhile, the surface wettability of the membrane can be analyzed from the water contact angle data, where a high membrane hydrophilicity is associated with low contact angle values [33].

**Figure 7:**
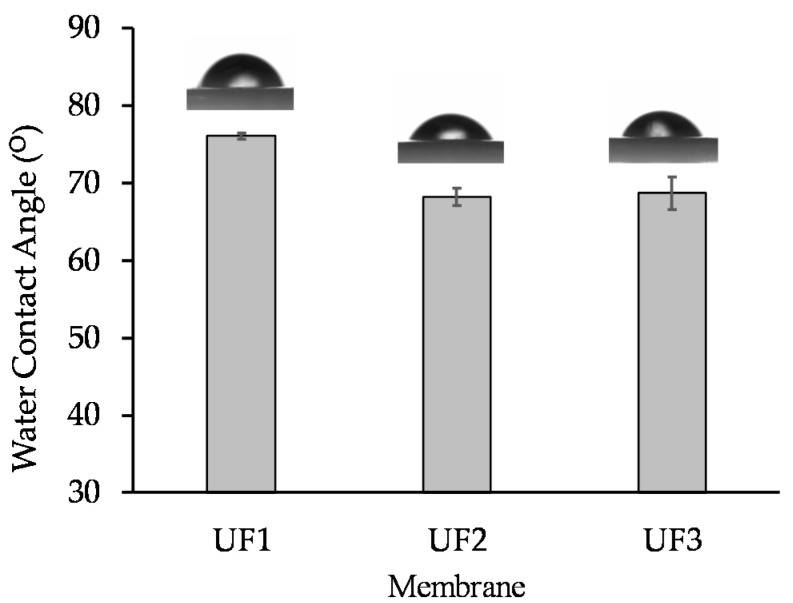
Water contact angle indicating membrane hydrophobic/hydrophilic properties.

The contact angle of water analysis results for all prepared membranes can be seen in Figure 7. The UF1 membrane (pristine PES) has the highest water contact angle (76.1 ± 0.4°). Blending 3 wt % of Plu with polymer solution (UF2) resulted in a membrane with a lower water contact angle (68.2 ± 1.1°), indicating higher hydrophilicity. Plu is a non-ionic surfactant formed by polyethylene oxide (PEO) and polypropylene oxide compounds (PPO) with a PEO- PPO- PEO block structure. The dominant EO group in Plu is a carrier of hydrophilic properties, lowering the UF2 surface contact angle. The addition of PO (UF3) did not cause a significant change in the hydrophilicity as it has a similar water contact angle (68.7 ± 2.1°) to that of UF2. During the phase inversion, the relative polarity of the PES, Plu and PO is very important, as this governs the membrane matric bulk structure. The non-polar Plu and PO are likely to move toward the water phase in the internal pore or to the top surface. The mobility of the non-polar compounds also contributes to the surface hydrophilicity. The water contact angle also relates to the surface roughness of the membrane. As can be seen in Figure 7, the water contact angle was lower at the higher membrane surface roughness (see Figure 4). It is worth noting that the hydrophilicity of the membrane surface can be attributed to the residual fraction of PO and Plu in the membrane matric. The impact may diminish when those chemicals leach out during the filtration operation, which can be a topic of a future work.

### 3.4. Membrane Strength

**Figure 8:**
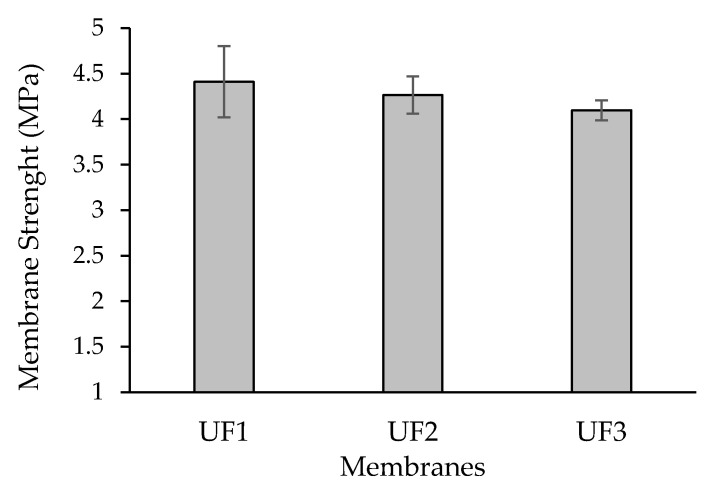
Membrane mechanical strength of the prepared PES membranes.

The profile of the tensile strength of the prepared membranes is presented in Figure 8. Mechanical strength indicates the ability of a membrane to withstand feed pressure during the filtration process. A tighter membrane structure and intermolecular molecules which are not spaced apart are directly proportional to excellent tensile strength. Figure 8 shows that the UF1 membrane had the highest tensile strength of 4.40 MPa. PES polymers are known to have strong mechanical properties. Modifying the polymer solution with Plu (UF2) and PO (UF3) only slightly decreased the tensile strength to 4.26 and 4.09 MPa, respectively, which is closely related to changes in the morphological structure dominated by finger-like macro voids, as shown in Figure 2. Therefore, it shows that the introduction of Plu and PO overall maintained the mechanical strength of the PES membrane. In addition, using One-way ANOVA with post-hoc Tukey HSD analysis, all prepared membranes are systematically significant for water contact value with *p* < 0.01 and systematically significant for tensile strength value (*p* < 0.05).

### 3.5. Anti-Bacterial Performances

**Figure 9:**
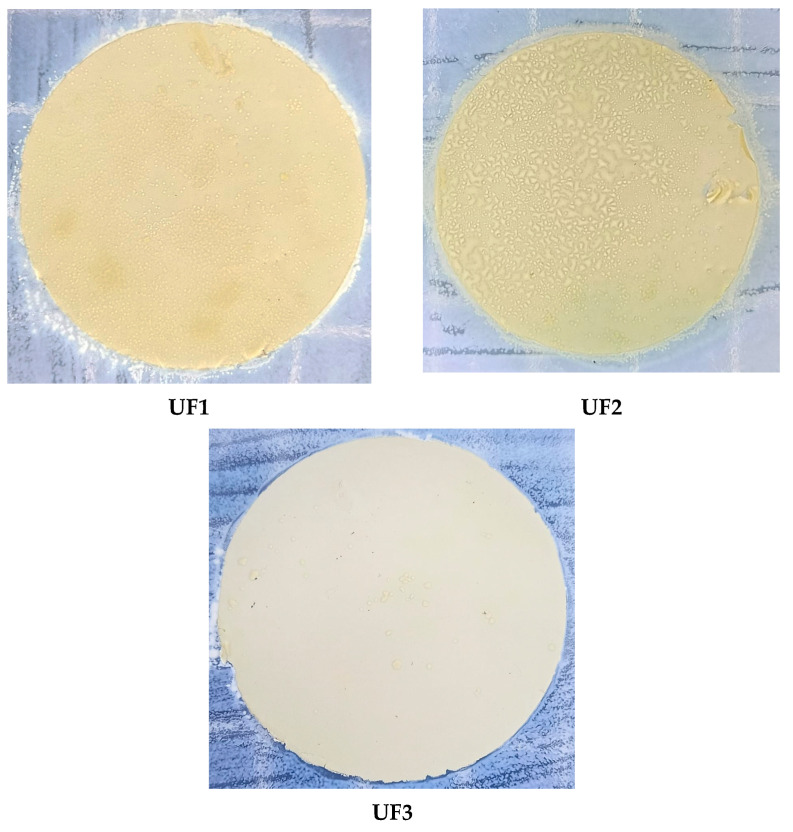
Anti-bacterial activity investigated by colony counting on membrane surface.

Since biofouling can reduce membrane performance permanently [34], the prevention of biofouling is highly needed. In this study, the qualitative and quantitative data of membrane anti-microbial performance are shown in Figure 9 and Table 2, respectively. The qualitative results show the visual performance of the growth colony on the membrane surface, while the quantitative data shows the number of colonies counted.

Figure 9 clearly shows that the colony of E. coli proliferates vigorously on the surface of the membrane without containing PO. The UF1 and UF2 membranes have similar results, in which the number of colonies counted were 390 and 360, respectively (Table 2). After the addition of 3% PO, the number of colonies is significantly reduced from 360 to 36 colonies. This is also supported by the visual performance of UF3 membrane surface in Figure 9, which shows the membrane surface has less colonies.

This anti-bacterial test proves that the phenolic compound of PO can mitigate the attachment of microbial cells and may disrupting the quorum sensing (QS) system of bacteria. QS is known to signal molecules that play roles in microbial cell-to-cell communication [35]. If the QS are interrupted by anti-microbial substances, then the number of microbial cells that grow in a current area can be reduced. In this study, PO acts as a quorum sensing inhibition (QSI) agent of the PES membrane.

### 3.6. Ultrafiltration Performance

Table 3 shows the water permeation results showing that the Plu addition to the polymer solution increased the membrane permeation (UF2) from 72.23 to 75.63 L m^−2^·h^−1^·bar, resulting from the presence of a large macro void and finger-like pore structures, as shown in Figure 2. However, the addition of PO (UF3) further increased the membrane flux to 85.87 m^−2^·h^−1^·bar. This significant permeation enhancement is believed to increase the hydrophilicity and antimicrobial properties induced by PO, which eventually leads to minimal fouling.

**Figure 10:**
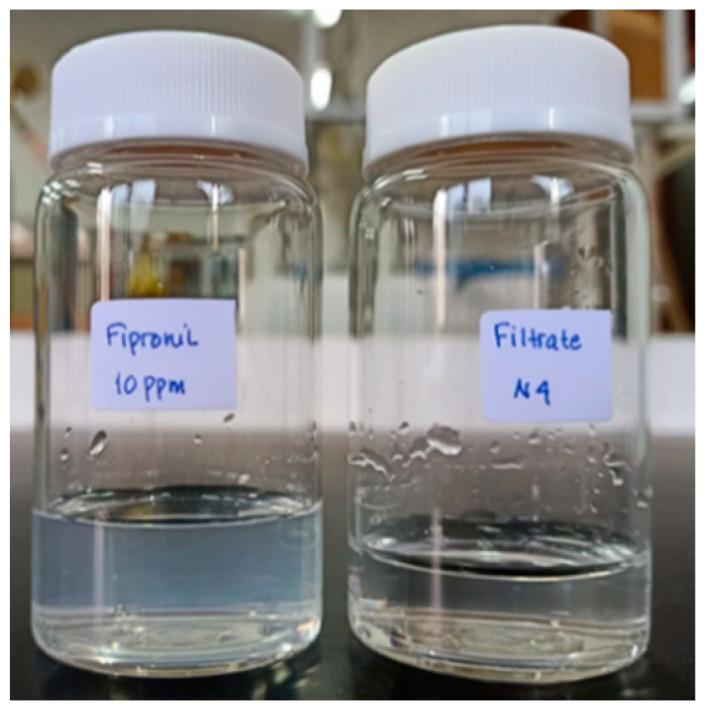
The visual performance of fipronil solution before and after filtration with UF3 membrane.

Table 4 also shows the effectiveness of the ultrafiltration process in removing fipronil pesticide solution. The highest pesticide rejection was obtained by the UF1 membrane, which was 85%. The rejection of pesticide solution using the UF2 and UF3 membranes was 84% and 75%, respectively. This is in agreement with other studies where the rejection is inversely proportional to the flux. A membrane with a high flux has a low rejection rate and vice versa. In this study, the highest rejection of fipronil pesticide was obtained through the UF1 membrane, which exhibits smaller pores than UF2 and UF3, leading to higher molecular retention. On the other hand, all prepared membranes are systematically significant for water permeability and fipronil rejection values with One-way ANOVA with post hoc Tukey HSD test *p* value less than 0.01 (Table 3). The permeate of fipronil filtration produced by the UF1 membrane filtration process is shown in Figure 10. The permeate is shown to be transparent, indicating that the membrane has retained the fipronil molecules.

Research on pesticide removal using membrane filtration has previously been reported by the team of Jung et al. By using commercial hollow-fiber nanofiltration membranes, pesticides can be removed from water samples in the range of 41.0% to 88.4% [36]. Another study on pesticide removal by membrane filtration was reported by Mukherjee et al. By using laboratory-scale polyamide RO membrane, researchers succeeded in removing pesticides in water in the range of 37.82% to 100% [37]. The pesticide rejection achieved in this study was generally high. This cannot be compared with the achievements of other studies because the types of membranes and pesticides used are different.

## 4. Conclusions

This study reveals the characteristics and performance of a modified hydrophobic PES membrane with pluronic and patchouli oil. The results establish that the incorporation of pluronic and patchouli oil in the PES membrane promoted the establishment of large macro voids and the number of finger-like structures by increasing length. The ultrafiltration test shows that the PES/Plu/PO membrane exhibited the highest flux and accomplished a rejection rate of fipronil pesticide solution up to 75%. The addition of Plu and PO in the membrane matrix enhanced the membrane hydrophilicity and the anti-bacterial property significantly.

## Figures and Tables

**Table 1:** Composition of the polymer solutions.

Membrane Code	PES	Plu	PO	DMF
	(wt %)	(wt %)	(wt %)	(wt %)
UF1	15	0	0	85
UF2	15	3	0	82
UF3	15	3	3	79

**Table 2:** Antimicrobial activity of the prepared membranes.

No	Membrane	Microbe Count	Unit
1	UF1	390	Colony
2	UF2	360	Colony
3	UF3	36	Colony

**Table 3:** One-way ANOVA with post hoc Tukey HSD test calculator.

Treatments Pair		Tukey HSD Q Statistic	Tukey HSD *p*-Value	Tukey HSD Inferfence
Membranes (UF1;UF2;UF3)	Water Contact	36.7491	0.0010053	** *p* < 0.01
	Tensile Strength	5.4501	0.0182416	* *p* < 0.05
	Water Permeability	25.9326	0.0010053	** *p* < 0.01
	Fipronil Rejection	36.2145	0.0010053	** *p* < 0.01

** Approach at set significance level (*p* < 0.01). * Below set significance level (*p* < 0.05).

**Table 4:** Prepared membrane performance in terms of permeability and selectivity.

No	Membrane	Water Permeability (L/m^2^.hr.bar)	Fipronil Rejection (%)
1	UF1	72.23	85.20
2	UF2	75.63	84.00
3	UF3	85.87	75.50

## Data Availability

Available on request to the corresponding author.
